# Glacial Waters Under Threat: Risk Assessment and Source Identification of Polychlorinated Biphenyls in Meili Snow Mountains, Southeastern Tibetan Plateau

**DOI:** 10.3390/toxics13050391

**Published:** 2025-05-13

**Authors:** Huawei Zhang, Yan Yao, Xinyu Wen, Rui Zhang, Rui Liu

**Affiliations:** 1 Faculty of Geography, Yunnan Normal University, Kunming 650500, China; gtjdxl@outlook.com; 2 College of Geography and Land Engineering, Yuxi Normal University, Yuxi 653100, China; yaoyan@yxnu.edu.cn (Y.Y.); lr870410@yxnu.edu.cn (R.L.)

**Keywords:** PCBs, risk assessment, ecological toxicological effects, driving factors, Meili Snow Mountains

## Abstract

Polychlorinated biphenyls (PCBs) are classified as persistent organic pollutants (POPs) due to their potential threat to both ecosystems and human health. The Tibetan Plateau (TP), characterized by its low temperatures, pristine ecological conditions, and remoteness from anthropogenic influences, serves as the investigation region. This study analyzed water samples from the temperature glacial watershed and employed the risk assessment method established by the United States Environmental Protection Agency (US EPA) to assess both carcinogenic and non-carcinogenic risks of PCBs in five age groups. The total concentrations of PCBs (∑_3_PCBs) varied from 738 to 1914 ng/L, with a mean value of 1058 ng/L, which was comparable to or exceeded levels reported in the surface water around the TP. Notably, the riverine sites located near the villages and towns exhibited the highest pollution levels. Our analyses indicated that glacier melting, long-range atmospheric transport (LRAT), reductive dechlorination processes, and various anthropogenic activities might be potential sources of PCB emission in the Meili Snow Mountains. According to the established national and international water quality standards, as well as toxic equivalency concentrations (TEQs) for dioxin-like PCBs (DL PCBs), the PCB concentrations detected in this study could result in serious biological damage and adverse ecological toxicological effects. However, the PCBs in all samples posed a negligible cancer risk to five age groups, and a non-carcinogenic risk to adults. These findings contribute valuable insights into the risks and sources of PCBs and may serve as a foundational reference for subsequent study of these compounds in the Meili Snow Mountains area of the southeastern TP.

## 1. Introduction

Persistent organic pollutants (POPs) in the environment have become eminent concerns around the global due to their potential carcinogenic effects [1,2]. POPs can reach high-altitude regions through long-range atmospheric transport (LRAT), resulting in their widespread distribution even in remote regions [3,4,5]. As a typical group of POPs, polychlorinated biphenyls (PCBs) are a family of hydrophobic chlorinated compounds characterized by high persistence and toxicity, bioaccumulative properties, and widespread distribution in the environment. PCBs are synthetic organic compounds characterized by a biphenyl structure that may incorporate between one and ten chlorine atoms. Based on their chemical structure and toxic properties, they are classified into two groups: dioxin-like PCBs (DL PCBs) (12 congeners: PCB77, 81, 105, 114, 118, 123, 126, 156, 157, 167, 169, and 189) and non-dioxin-like PCBs (non-DL PCBs) (197 congeners) [6,7]. Due to their persistence, high toxicity, and bioaccumulation ability, the production and the application of PCBs have been restricted for nearly 50 years [8]. However, some studies have found that PCBs are widely present in water, dust, sediment, air, vegetable, organisms, and even human tissues [6,9,10,11,12]. Rivers and glaciers can act as reservoirs for PCBs, and glacier melting can release PCBs into rivers that are used for drinking water and make them bioavailable, thereby posing serious threats to both ecological integrity and human health. Previous studies have demonstrated that PCBs can accumulate in fatty tissues and disrupt various bodily systems, including the endocrine, nervous, reproductive, and immune systems [13]. They also pose cancer risk and can be fatal [13]. Additionally, non-DL PCBs and their metabolites may cause neurotoxic effects, inducing developmental and behavioral problems, by disturbing thyroid hormone levels, interfering with cellular signaling, disturbing in neurotransmitter levels, and altering gene expression [14,15].

PCBs originate from historical use and improper disposal activities [16,17]. PCBs can also be released during combustion and industrial thermal processes, or unintentionally produced as by-products in some manufacturing industries [16,17,18,19,20]. PCBs enter the aquatic environment through pathways including atmospheric transport and deposition, runoff from terrestrial sources, and industrial and municipal wastewater discharge [13,21,22,23,24]. Upon entering the aquatic environment, PCBs progressively migrate between various phases, eventually leading to water pollution and indirect toxicological effects on the ecosystem. Moreover, the aquatic environment constitutes a major part of our ecological systems and water resources; therefore, its safety is directly related to human health.

Due to low temperatures and pristine ecological conditions, the Tibetan Plateau (TP) is commonly designated as the “Third Pole” of the Earth [25], characterized by its remoteness and inaccessibility. Additionally, the TP is the origin for many major rivers in Asia and is a vital water resource for about one-sixth of the global population [26]. However, the TP is located at a low latitude and is surrounded by the rapidly industrializing nations of South Asia and Southeast Asia. Semi-volatile POPs from these sources can undergo LRAT and subsequently experience cold-trapping in the TP regions [11,27]. In glacial watersheds, the accumulation of POPs in the aquatic environment is the result of an interaction between the atmosphere and these pollutants that occurs through falling snow and precipitation, leading to the environmental and ecological integrity of the TP being affected by anthropogenic activities from these sources. As a typical representative of the temperate glacial watersheds in southeastern TP, the Meili Snow Mountains (98°30′-98°46′ E, 28°10′-28°41′ N) constitute a continuous mountain range and serve as an integral part of the Hengduan Mountains. The area with the highest elevation is Kawagebo Peak, at 6740 m above sea level. This mountain range is vital for supplying agricultural irrigation and domestic water to populations residing in downstream areas; thus, it plays a critical role in the ecological health and well-being of these communities. The Meili Snow Mountains are also in proximity to the Indian subcontinent, which is characterized by its large-scale manufacture and use of POPs [28].

In the TP region, investigations into POPs have predominantly focused on PAHs. However, there is a notable lack of environmental monitoring data on PCBs, and, to our knowledge, no extensive studies have been conducted to assess PCB pollution. Therefore, this study was initiated to analyze the concentrations, distribution, and compositional patterns of PCBs in the surface waters from the Meili Snow Mountains located in the southeastern TP. Additionally, a preliminary ecological and health risk assessment was preformed to elucidate the extent of PCB pollution and its associated risks in this particular region.

## 2. Materials and Analytical Methods

### 2.1. Sample Collection

**Figure 1:**
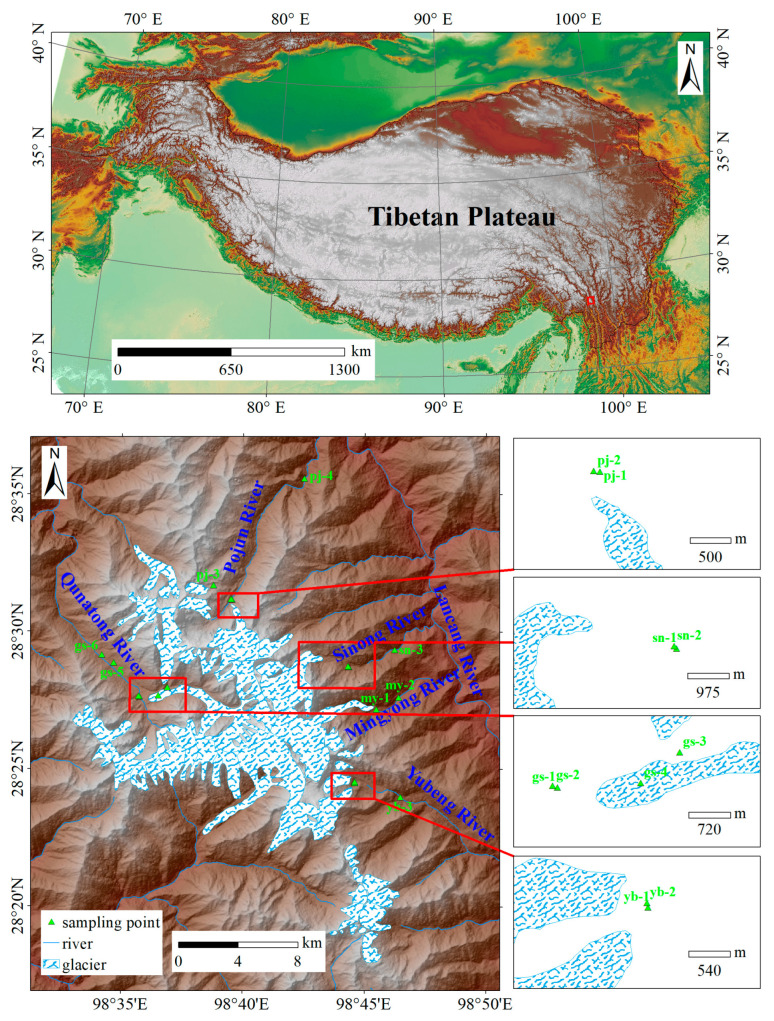
The locations of the water sampling points in the Meili Snow Mountains.

Glacial meltwater and river water samples were collected from the Meili Snow Mountains in October 2023 (Figure 1). Five river water samples were collected from the downstream regions of rivers originating from glacial meltwater across various altitudinal gradients, and eighteen water samples were collected and analyzed in total. The sampling points were determined by global navigation satellite system equipment, and the comprehensive details are provided in Table S1. Each sample was stored in a clean 2 L low-density polyethylene bottle (Thermo Scientific, Waltham, MA, USA) that was pre-cleaned with ultra-pure water, methanol, and acetone to ensure pollution-free conditions, then washed with water from a particular sampling point. The collected samples were then filtered through 0.45 μm membranes to remove particulate matter and transferred to new low-density polyethylene bottles. The samples were preserved at −4 °C during transport to the analytical laboratory at the Beijing Institute of Geology of Nuclear Industry, where they were subsequently stored at −18 °C until analysis.

### 2.2. Chemicals and Reagents

All chemicals used for sample processing and analysis were of analytical, liquid chromatography, or pesticide residue grade, and were obtained from Wako Chemical (Osaka, Japan) and Tan-Mo Technology Co., Ltd. (Changzhou, China). Deionized water, with a resistivity of 18 MΩ·cm, was obtained from a Milli-Q water purification system (Merck KGaA, Darmstadt, Germany). Florisil, with a particle size of 60–100 mesh, was activated in an oven at 130 °C for a duration of 24 h.

### 2.3. Sample Pretreatment

The water samples were thoroughly agitated, and 1 L was precisely measured into a separatory funnel. Substitute standard solution was added to each water sample and thoroughly mixed. The pH of the water samples was adjusted to a range of 5–9 with hydrochloric acid. Then, 30 g sodium chloride and 50 mL n-hexane were sequentially added to the water samples, and the mixture was shaken for 5 min and allowed to stand for stratification until the aqueous phase and organic phase separated. The extraction process was repeated twice. The sample was dried through a drying column and then concentrated to 10 mL. Subsequently, the solution was concentrated to 1 mL, using sulfuric acid to purify it first. Then, a Florisil column was used for purification, and the eluent was further concentrated to less than 1 mL. An internal standard solution was added and adjusted to 1 mL with n-hexane. The internal standards of PCBs consisted of PCB77-d_6_, PCB156-2′,6,6′-d_3_. Finally, the prepared samples were stored at −4 °C until analysis.

### 2.4. Gas Chromatography-Mass Spectrometry Analysis

The pretreated sample was analyzed using gas chromatography-mass spectrometry (GC-MS, Clarus 600, 8547, Perkin Elmer, Waltham, MA, USA) with electron ionization. Helium gas was used as the carrier gas at a constant flow rate of 1.2 mL/min during the analysis. Chromatographic separation was conducted using a quartz HP-5MS capillary column with dimensions 30 m × 0.25 mm × 0.25 μm (Agilent Technologies, Santa Clara, CA, USA). Each sample was injected at a volume of 1.0 μL in splitless injection mode. The injector temperature was maintained at 280 °C, and the electron impact ionization voltage was set to 70 eV, with the ion source temperature set to 280 °C. The temperature program for the oven was as follows. The column was initially kept at 120 °C for 1 min, subsequently increased to 180 °C at a rate of 20 °C/min, and finally increased to 280 °C at a rate of 10 °C/min, with a hold time of 20 min. Identification of the PCB congeners was achieved by electron impact spectrometry in selected ion monitoring mode. During GC-MS analysis, the quantification of PCBs was performed by establishing a correlation between the analyte concentration passing through the detector and the corresponding detector response, which was based on the peak area. The peak height during mass spectrometry potentially fluctuated in relation to the flow rate of the mobile phase.

The 18 PCB congeners quantified were PCB28, PCB52, PCB77, PCB81, PCB101, PCB105, PCB114, PCB118, PCB123, PCB126, PCB138, PCB153, PCB156, PCB157, PCB167, PCB169, PCB180, and PCB189.

### 2.5. Quality Assurance and Quality Control

The limit of detection (LoD) for the target analyte was determined using a specific method: a blank sample was subjected to injection, followed by the repeated injection of 10 needles. Then, the standard deviation of the integral peak area at the designated retention time for the target substance was calculated, and the corresponding content was calculated with a standard deviation value of 3 as the detection limit. The certified material used in the analytical process consisted of a mixture of 18 PCBs in toluene (lot number: 21050127, part number: 80010GN, Tan-Mo Technology Co., Ltd., Changzhou, Jiangsu, China). The recovery rate and precision data are presented in Table S2.

### 2.6. Risk Assessment Methods

In this study, we used hazard quotient (HQ) and incremental lifetime cancer risk (ILCR) as evaluation indexes to conduct carcinogenic and non-carcinogenic risk assessments for PCBs, as these are recommended by the US EPA [9,29,30,31]. The calculation formulas are as follows:

*HQ* = ∑*C_i_* × *IR* × *EF* × *ED* × 10^−6^/(*BW* × *AT* × *RfD*) (1)

*ILCR* = *CSF* × ∑(*C_i_* × TEF*_i_*) × *IR* × *EF* × *ED* × 10^−6^/(*BW* × *AT*) (2)

where HQ is the non-carcinogenic risk of PCBs; ILCR is the carcinogenic risk of PCBs; *C_i_* is the concentration of PCBs congeners (ng/L); and TEF*_i_* is the corresponding toxic equivalency factor. In this study, the TEF values of DL-PCBs, including PCB81 and PCB105, with 2,3,7,8-tetrachlorodibenzo-p-dioxin are 0.0003 and 0.00003 [32], respectively. CSF is the carcinogenic slope factor of PCBs, with a value of 2 (kg·d)/mg [30,33]; RfD is the noncarcinogenic reference dose of PCBs, quantified as 2.3 × 10^−5^ mg/(kg·d) [34]. Other parameters are shown in Table 1.

Lifetime can be divided into five age groups: 0–1 yrs (infants), 1–3 yrs (toddlers), 3–10 yrs (children), 10–20 yrs (teenagers), and 20–75 yrs (adults). Detailed information on the primary exposure parameters is presented in Table 1.

According to the US EPA guidelines [30,35] and previous studies [6,36], an ILCR of less than 10^−6^ indicates a safe level of risk, whereas an ILCR value ranging from 10^−6^ to 10^−4^ represents low health risk. If an ILCR value is greater than 10^−4^, there is a significant potential risk. A value of HQ that is less than 1 indicates that the daily exposure dose may have no detrimental effects on human health [6,35].

### 2.7. Air Masses Backward Trajectories

To assess the origin of air masses arriving at the investigation area, 5-day backward air trajectories were computed for the two years prior to sampling, from October 2021 to September 2023, by the National Oceanic and Atmospheric Administration (NOAA) Hybrid Single-Particle Lagrangian Integrated Trajectory (HYSPLIT) model. These trajectories were generated with arrival heights of 1000 m and cluster analysis was performed at three-month intervals.

## 3. Results

### 3.1. Occurrence and Composition of PCBs

**Figure 2:**
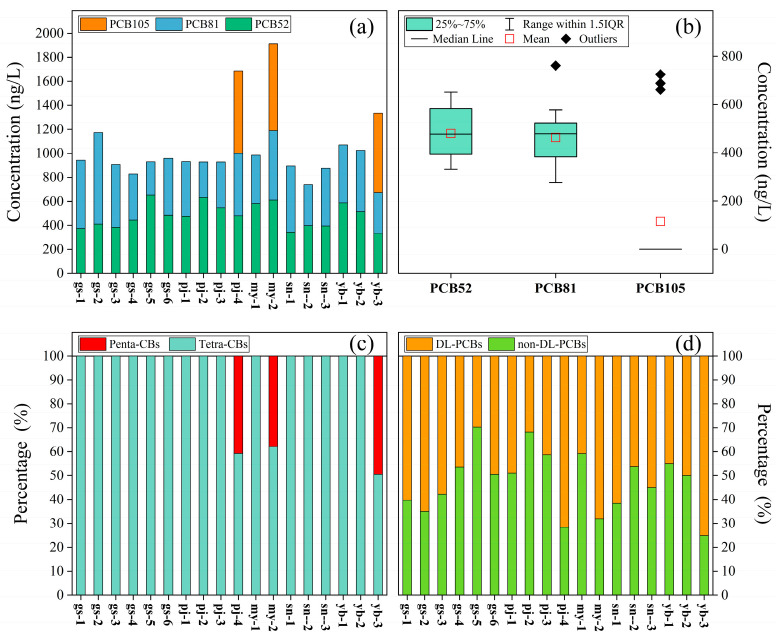
The concentrations of individual PCBs (**a**), a box line diagram showing PCB52, PCB81, and PCB105 (**b**), and concentration composition pattern comparisons of PCBs (**c**) and DL and non-DL PCBs (**d**) at all sampling points.

The data on the PCBs detected in all samples are shown in Figure 2a. Additionally, more descriptive statistics data on 18 PCB concentrations are presented in Table S3. The analysis revealed that PCB52 and PCB81 were present in all samples, whereas PCB105 was exclusively identified in pj-4, my-2, and yb-3 at elevated concentrations. The concentrations ranged from 332 to 653 ng/L, with a mean value of 479.8 ng/L for PCB52; 276 to 762 ng/L, with a mean value of 463 ng/L for PCB81; and a LoD of 725 ng/L with a mean value of 115.3 ng/L for PCB105, respectively. The concentration of total PCBs (∑_3_PCBs) varied between 738 and 1914 ng/L, with a mean value of 1058.1 ng/L. The highest ∑_3_PCB concentration appeared at my-2 (1914 ng/L), followed by pj-4 (1688 ng/L) and yb-3 (1335 ng/L), while the lowest was at sn-2 (738 ng/L). Moreover, as shown in Figure 2b, the concentration of PCB52 demonstrated the most substantial variation, followed by PCB81. The significant variability in the PCB52 concentration indicated that its spatial distribution was more heterogeneous in the Meili Snow Mountains area.

The proportion of PCB congeners to ∑_3_PCBs is shown in Figure 2c. It can be seen that PCB patterns were predominated by low-chlorine (1–4 Cl) PCBs, such as tetra-CBs (89.1%), while high-chlorine (5–10 Cl) PCBs, such as penta-CBs (10.9%), were less prominent. There were no obvious differences in the proportion of low-chlorine PCBs, with the exception of pj-4, my-2, and yb-3, indicating that there was no point source pollution input for these low-chlorine PCBs in the Meili Snow Mountains area, and their distribution might be influenced by the atmospheric deposition and glacier melting transport.

The composition of DL and non-DL PCBs is shown in Figure 2d. With the exception of gs-1, gs-2, gs-3, pj-4, my-2, sn-1, and yb-3, the non-DL PCBs were the predominant PCBs. In addition, non-DL PCBs exhibited a wide range of changes. The highest concentration of DL PCBs was in the rural river water (yb-3), followed by pj-4, whereas glacial meltwater exhibited relatively high concentrations of non-DL PCBs. And the highest proportions of non-DL and DL PCBs in water samples were at gs-5 and yb-3, respectively.

### 3.2. Health Risk Values

**Figure 3:**
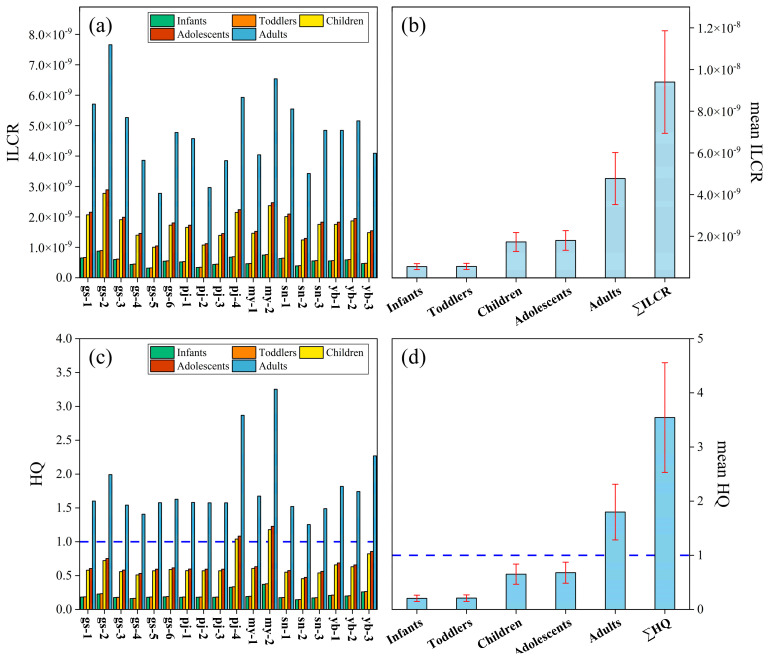
A comparison of potential health risks occurring through the ingestion of the surface water. Carcinogenic exposure risks (ILCR (**a**) and mean ILCR (**b**)) and non-carcinogenic exposure risks (HQ (**c**) and mean HQ (**d**)) are shown.

The values of both carcinogenic and non-carcinogenic risk are presented in Figure 3. The carcinogenic and non-carcinogenic risks for adults were the highest among the five age groups. The mean ILCR values were 5.45 × 10^−10^ for the infant group, 5.56 × 10^−10^ for the toddler group, 1.73 × 10^−9^ for the child group, 1.80 × 10^−9^ for the adolescent group, and 4.77 × 10^−9^ for the adult group (Figure 3a). The ILCR values were less than 1 × 10^−6^, indicating a negligible cancer risk. The lifetime cancer risk values (∑ILCR) ranged from 5.47 × 10^−9^ to 1.51 × 10^−8^, with a mean value of 9.40 × 10^−9^ (Figure 3b). After 70 years of continuous exposure through ingestion of the surface water, the cancer risk was still negligible (Figure 3b). The mean HQ values were 0.21 for the infant group, 0.21 for the toddler group, 0.65 for the child group, 0.68 for the adolescent group, and 1.80 for the adult group (Figure 3c). The HQ values were lower than 1, with the exception of the adult group, indicating no detrimental effects for the other four age groups. The lifetime non-carcinogenic risk values (∑HQ) ranged from 2.47 to 6.41, with a mean value of 3.54 (Figure 3d). After 30 years of continuous exposure, the non-carcinogenic risk significantly increased.

### 3.3. Ecotoxicological Risk Values

**Figure 4:**
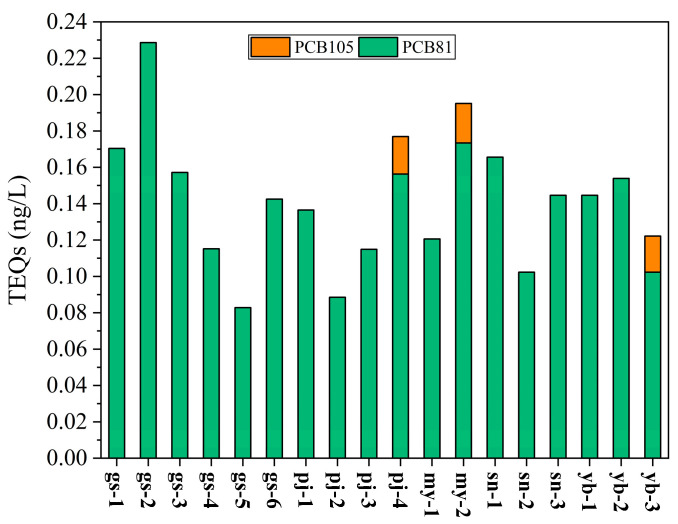
The TEQs of DL-PCBs in the surface water of the Meili Snow Mountains.

Considering the toxicity and bioaccumulation properties of PCBs, and the vulnerable ecological environment of the TP, it is crucial to evaluate the potential ecological risk of DL-PCBs in this region. In this study, we estimated the toxic equivalency concentrations (TEQs) by multiplying the concentration of each DL-PCB by TEF [33]. The TEQ of each DL-PCB congener varied from 0 to 0.17 ng/L (Figure 4). The sum of the TEQs (∑TEQs) for the DL-PCBs detected in all samples ranged from 0.09 to 0.23 ng/L, with a mean value of 0.14 ng/L (Figure 4). The results showed that the ∑TEQs varied greatly and were mainly contributed to by PCB81. The highest ∑TEQ value was at gs-2, followed by my-2 and pj-4, while the lowest appeared at gs-5, which was a somewhat different pattern to that observed with PCB52 as the primary indicator PCB.

### 3.4. Air Masses Backward Trajectories

**Figure 5:**
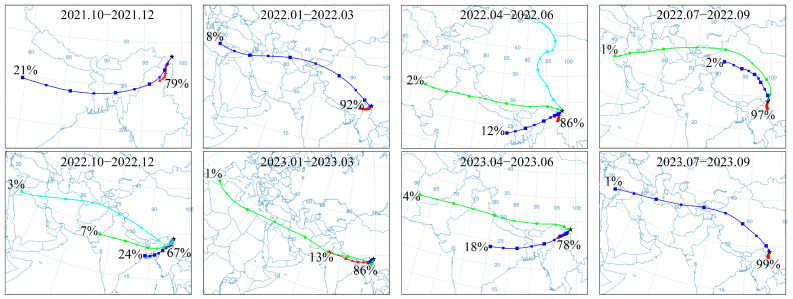
A cluster analysis of the 5-day backward trajectories of air masses in the Meili Snow Mountains from October 2021 to September 2023. The colored lines present different clusters.

Figure 5 shows the clustering results of the backward trajectories over the Meili Snow Mountains during the two years prior to the sampling. In the two years prior to the sampling, during the period from October to December, about 70% of the air masses in the Meili Snow Mountains came from northern Myanmar, and 20% came from northern India and Bangladesh; from January to March, about 90% of the air masses in the Meili Snow Mountains came from northern Myanmar; from April to June, 89% of the air masses in the Meili Snow Mountains came from northern Myanmar, and 20% came from northern India and Bangladesh; and from July to September, over 95% of the air masses in the Meili Snow Mountains came from northern Myanmar. Therefore, these air masses were primarily influenced by the Indian monsoon and westerly winds.

## 4. Discussion

### 4.1. Comparison with Global Related Studies

**Figure 6:**
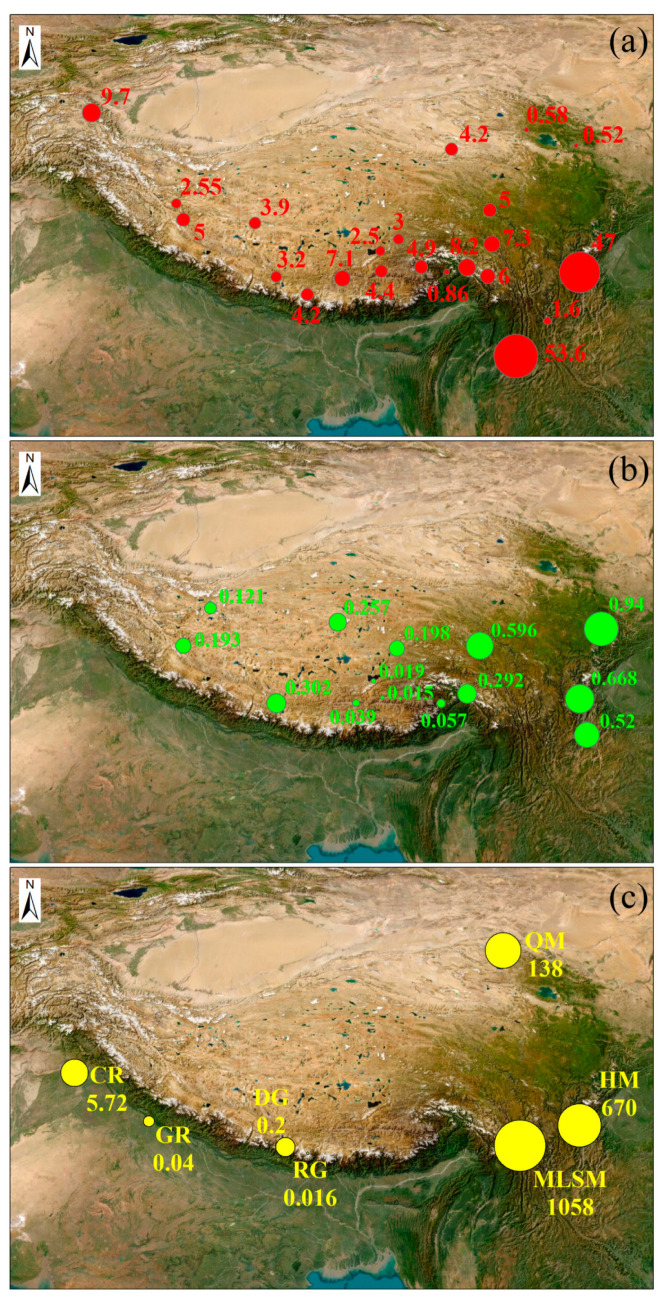
The mean ∑PCBs of different samples from different regions around the Tibetan Plateau—air samples (pg/m^3^) (**a**), soil samples (pg/g) (**b**), and water samples (ng/L) (**c**).

Figure 6 shows related research on PCBs using samples from around the TP, including air, soil, and water. In the air of the southeastern TP (Figure 6a, Table S4 [26,37,38,39,40,41,42,43]), the concentration of PCBs is relatively high, reaching 53.6 pg/m^3^ in the Tengchong mountainous area [42] and 47 pg/m^3^ on Gongga Mountain [41], while other regions have lower concentrations. In the soils of the eastern TP (Figure 6b, Table S5 [44,45,46,47,48]), the concentration of PCBs is relatively high, with the highest concentration in the Ruoergai grassland and wetland, reaching 0.94 pg/g [45], followed by the Sichuan mountainous areas, with concentrations of 0.668 pg/g and 0.52 pg/g [44]. In the surface soil of the Tibet region, the concentrations of PCBs in different types of soil are as follows: sub-alpine scrub-meadow (Nangqen, Yushu, China, 0.596 pg/g), mountain shrubby steppe (Zhongba, Shigatse, China, 0.302 pg/g), lpin-burozems (Bomi, Linzhi, China, 0.292 pg/g), alpine steppe (Linge Co, Changdu, China, 0.257 pg/g), alpine meadow (Naqu, China, 0.198 pg/g), sub-alpine desert (Gar, Ali, China, 0.193 pg/g), and alpine desert (Hoh Xil, Ali, China, 0.121 pg/g) [48]. Atmospheric pollutants can be transferred to the surface water through dry and wet deposition, while pollutants in the soil can be introduced into the surface water through runoff, leading to the enrichment of pollutants within these aquatic systems. Compared to studies on PCBs in the water around the TP, the concentration of PCBs in this study is the highest, reaching 1058 ng/L (Figure 6c, Table S6 [10,49,50,51,52]). This indicates that the pollution level of PCBs in the Meili Snow Mountains area is very high and requires urgent attention. Meanwhile, anthropogenic activities are relatively frequent and intense, particularly compared to the Gongga Mountain and Qilian Mountain areas [10].

### 4.2. Factors Influencing PCB Distribution

#### 4.2.1. Local Environment Driven Pattern

**Figure 7:**
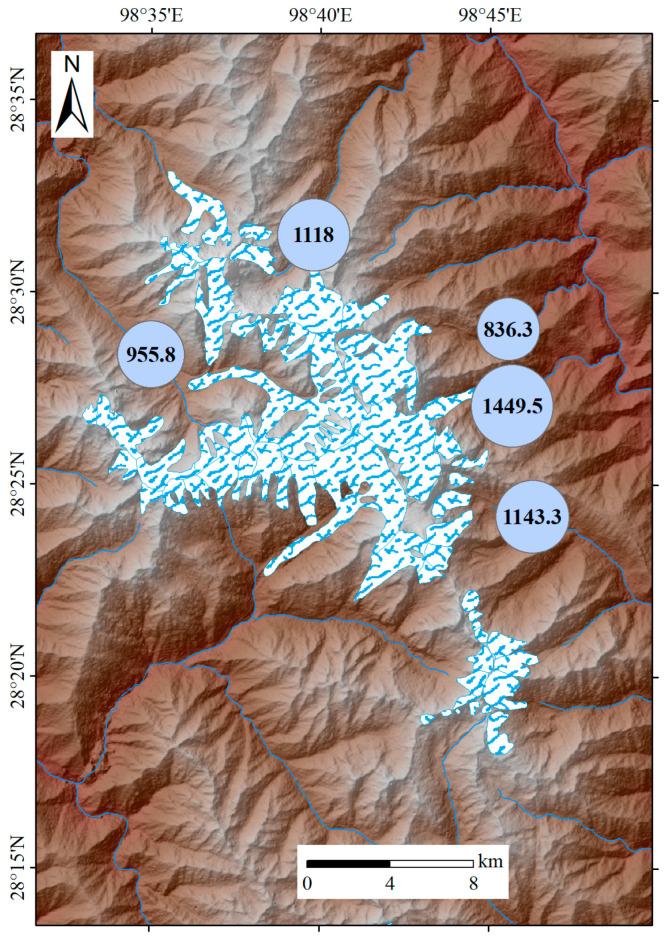
The mean ∑_3_PCB (ng/L) distributions of water samples in different watersheds.

**Figure 8:**
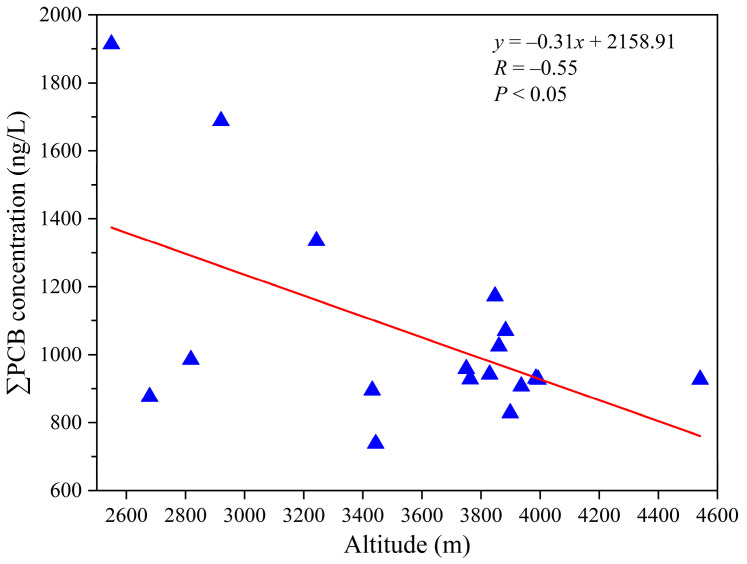
The relationship between ∑_3_PCBs and altitude.

Figure 7 displays the mean ∑_3_PCB concentrations in water samples from five different watersheds, including the Qunatong, Pojun, Mingyong, Sinong, and Yubeng River watersheds (Table S1). The highest mean ∑_3_PCB concentration appeared at the Mingyong River watershed (1449.5 ng/L), followed by the Yubeng River watershed (1143.3 ng/L) and the Pojun River watershed (1118 ng/L), while the lowest was at the Sinong River watershed (836.3 ng/L). The three regions with relatively high mean ∑_3_PCB concentrations are located in proximity to densely populated villages and towns, and also serve as tourist destinations. Consequently, they are particularly vulnerable to pollutants originating from local anthropogenic sources. Additionally, previous studies have demonstrated that among the various factors affecting POP concentrations, such as distance from sources, latitude, altitude, and other variables [53,54], altitude is the biggest determinant [10]. This study indicates that ∑_3_PCB levels exhibit a decreasing trend with increasing altitude (Figure 8), which might be attributed to the enhancement of local convergence and enrichment mechanisms. Variations in atmospheric pressure, the rates of dry and wet deposition, and the distance from pollution sources can affect the ∑POP concentration along with changes in altitude [10,53]. Generally, the low-altitude regions located in densely populated villages and towns are impacted by various sources of pollution. Anthropogenic activities, such as the combustion of wood and coal for heating, vehicular transportation, untreated waste water discharge, waste incineration, industrial processes, and the disposal of electronic waste [3], contribute to the introduction of PCB pollutants into the water-vapor interface, thereby exerting a substantial effect on aquatic ecosystems. Therefore, it is essential to comprehend the influences of local anthropogenic activities on the release of PCBs into the environment.

#### 4.2.2. Regional Atmospheric Origins

LRAT is an important contributor to the presence of POPs in remote regions [26]. The Indian monsoon is the dominant climatic driver affecting the Meili Snow Mountains (Figure 5), leading to the inference that monsoon transport is likely the primary source of POPs in the atmosphere. Additionally, PCB concentrations in the aquatic environment are directly associated with the atmospheric deposition of PCB pollutants [3,55]. Notably, atmospheric PCB levels around the TP have exhibited a significant correlation with the cyclical patterns of the Indian monsoon, with elevated levels observed during the monsoon season and reduced levels during the non-monsoon season [12,26,55]. Low-chlorine PCBs, especially tetra-CBs, have been identified as the predominant form in the atmosphere of the TP [37]. Furthermore, previous studies have indicated that tetra-CBs are also the dominate type at rural sites in India [56] and in the equatorial region over the Indian Ocean [57], thereby confirming the influence of this source region on the atmospheric composition of the TP. The predominance of low-chlorine PCB congeners in this study, especially tetra-CBs, provides additional evidence that PCBs can reach the TP through atmospheric transport mechanisms. Moreover, tetra-CBs have a higher potential for LRAT [37]. The PCB composition in this study exhibits similarities to those in waters of the eastern TP [10] and in seawater samples from the North Atlantic and Arctic oceans [3,58,59]; however, it differs from the compositional patterns in marine waters at temperate latitudes, where high-chlorine PCBs predominate [60]. This discrepancy can be attributed to the solubility and volatility of PCBs, which decrease with an increasing number of chlorine atoms. Low-chlorine PCBs exhibit a higher tendency to volatilize into the atmosphere, subsequently accumulating in the aquatic environment through dry and wet deposition, whereas high-chlorine PCBs are less diffusible and are more efficiently deposited in proximity to their sources [3,61,62]. One portion of the air mass originates from the industrialized and densely populated regions of northern India and Bangladesh, while another portion is derived from northern Myanmar, which is known for slash-and-burn agricultural practices, serving as a source of PCBs. The effective removal of atmospheric pollutants requires substantial precipitation [63]. The southeastern TP region receives considerable precipitation during the monsoon season, with an average annual precipitation of approximately 800 mm [64]. The results of this study suggest that rain scavenging likely plays a crucial role in the elevated PCB concentrations in the aquatic environment. Furthermore, climate change could result in increased precipitation in the future, thereby facilitating the transfer of PCBs from the atmosphere to the aquatic environment [3]. Therefore, it is projected that the southeastern TP may experience an increased influx of PCBs from the atmosphere. Apart from wet deposition, atmospheric PCBs may also be transported through dry deposition [61], with diffusive gas exchange potentially playing an important role in this transport mechanism [26]. Nevertheless, assessing the relative significance of these processes remains a considerable challenge at present.

#### 4.2.3. Impact of Melting Glaciers

Climate warming can facilitate the rapid melting of glaciers, which has been recognized as a major contributor to runoff rivers originating from glacial regions [65]. During the summer, pollutants accumulated in glaciers can be effectively released into runoff rivers. This study demonstrates that glacier meltwater plays an important role in the transport of PCBs, as evidenced by their elevated concentrations in water samples from glacier-fed rivers (Figure 1 and Figure 2a). Previous studies have demonstrated that the composition of POPs in glacier meltwater closely resembles that in river water from the same watersheds [10]. Additionally, high-molecular weight POPs tend to deposit during condensation processes, resulting in relatively higher concentrations of low-molecular weight POPs in high-altitude regions [66], which are supported by the composition and concentration of PCBs in the surface water of the Meili Snow Mountains (Figure 2a). The influence of glacier meltwater on PCB pollution in river water was particularly pronounced in the Qunatong River, where variations among the sampling points were observed, especially in areas affected by glacial proximity compared to those further away (Figure 1 and Figure 2a). It is evident that the rivers receiving glacier meltwater accumulate the pollutants within the glacier, leading to elevated ∑_3_PCB levels in the river water. The accumulation of pollutants from previous periods on glacier surfaces will be exacerbated by the effects of climate warming [3,65]. Furthermore, pollutants stored in glaciers, snow, and ice particulates are released into the runoff rivers during melting processes [67]. Therefore, we infer that glacier melting may serve as a source of PCBs, which is supported by the presence of POPs in seawater from Hornsund [3] and in the surface water from eastern TP [10]. Moreover, due to lipophilicity, the solubility of PCBs decreases with an increase in molecular weight [10,68]. Therefore, low-chlorine PCBs exhibit greater solubility in water. Tetra-CBs demonstrate considerable environmental persistence and greater LART potential [37], which is consistent with the prevalence of PCB52 and PCB81 observed in all samples from the Meili Snow Mountains (Figure 2a).

### 4.3. Potential Sources

The presence of PCBs in the environment may be affected by environmental media, the stability of different PCB congeners, and the characteristics of their emission sources, resulting in the absence of known proportions of specific sources of PCBs, especially in cases involving multiple sources [69]. Therefore, the identification of PCBs’ origins poses considerable difficulties, primarily due to their multi-source characteristics and the lack of specific molecular diagnostic ratios. However, the specific composition of PCBs can provide information for potential sources, as different PCB congeners are associated with distinct emission sources. For example, PCB52 is recognized as an indicator of transformer oil and Aroclor 1242 [16,70]. In the US, PCB105 is typically found in elevated concentrations within the Aroclor series [71], while PCB 81 is primarily associated with solid waste incineration processes [16]. Additionally, PCB52 and PCB81 are likely by-products of coal and wood combustion, as well as various industrial thermal processes [72]. High-chlorine PCBs are easily adsorbed to sediments and particulate matter upon entering aquatic environments, which is attributed to their distinctive physical and chemical properties. Under anaerobic conditions, the reductive dechlorination of high-chlorine PCBs by specific microorganisms may also contribute to the elevated levels of low-chlorine PCBs in water [73,74,75]. Overall, the PCB52 and PCB81 in this study may originate from the combustion of coal and wood, solid waste incineration processes, commercial PCB mixtures, and reductive dechlorination processes. Furthermore, the disposal of electronic devices may also be a primary source of PCB52. Previous studies have demonstrated that the emission sources of PCBs are complex and are significantly associated with various anthropogenic activities within terrestrial ecosystems [76,77]. Therefore, it is imperative to conduct further investigations to elucidate these sources in subsequent studies.

### 4.4. Ecotoxicological Risk

PCBs are hazardous synthetic compounds that can disrupt endocrine and immune systems, affect reproductive health, and increase cancer risk in vertebrates [13]. A previous study has reported that the long-term viability of over 50% of the global killer whale population is threatened by PCB-mediated effects on their reproduction and immune function [78]. Therefore, it is crucial to evaluate the ecological and human health risks of PCBs in this region. However, due to the lack of established environmental quality standards in China, our results can only be compared with those published by other nations and international organizations. The ∑_3_PCB levels of all samples, ranging from 738 to 1914 ng/L, were below the criterion maximum concentration (CMC) for water quality as defined by the US NOAA, which is set at 20 ng/L for inland waters and 10,000 ng/L for coastal waters [19]. This indicates that organisms exposed to these aquatic conditions face minimal or negligible risk. However, the Σ_3_PCB level exceeded the criterion continuous concentration (CCC) thresholds established by the US EPA and the US NOAA, which are intended to protect both aquatic ecosystems and human health from the risks associated with chronic exposure to dissolved PCBs. The CCC values are set at 14 ng/L for inland waters and 30 ng/L for coastal waters [29]. Additionally, the observed levels significantly exceeded the Chinese national environmental quality standards for surface water [79], which establish a maximum permissible limit of 20 ng/L. Consequently, the surface water in the Meili Snow Mountains is heavily polluted with PCBs, implying that the chronic toxicity of the ∑_3_PCB levels in its aquatic ecosystems have adverse implications for ecological integrity.

The presence of POPs in aquatic environments has negative effects on phytoplankton and zooplankton, including organisms like algae, diatoms, water fleas, and scud [80]. Aquatic organisms are susceptible to the effects of dissolved PCBs, especially their biological and toxicological impacts [13,21], which have caused great concern. This study aimed to assess the ecotoxicological effects of DL-PCBs in surface water by calculating their TEQs. The findings indicate that the TEQ of each detected DL-PCB congener exceeded the environmental quality standard of 1 pg/L set by the Japanese government [81]. Additionally, the ∑TEQs in all samples exceeded the maximum contaminant concentration of 30 pg/L established by the US EPA [31], although these levels remained below the maximum contaminant level of 8 ng/L defined by the environmental quality standards for water in China [21]. Consequently, the observed levels of DL-PCBs are likely to pose a threat to both water quality and aquatic organisms in the TP region. Moreover, the bioavailability of PCBs may enhance their accumulation through biomagnification processes, potentially resulting in biological impairments in higher organisms, including humans.

### 4.5. Health Risk Assessment

The toxicological effects of DL PCBs are primarily mediated by the aryl hydrocarbon receptor (AhR). In contrast, non-DL PCBs can induce a variety of responses through different toxicological mechanisms that do not involve AhR [82]. It should be realized that individual congeners may exert themselves through multiple mechanisms, resulting in a range of effects including carcinogenicity, immunotoxicity, and adverse effects on reproductive, developmental, and endocrine functions [83,84]. Consequently, PCBs have exhibited both carcinogenic and non-carcinogenic effects. An assessment of five age groups revealed that the adults exhibited the highest carcinogenic risk, while infants presented the lowest. The ILCR values for the five groups were far below 10^−6^, indicating that there was no cancer risk. Non-carcinogenic risk assessments indicated that the adults faced the highest risk of developing non-carcinogenic effects, whereas infants exhibited the lowest. Excluding the adult group, the non-carcinogenic risk values for the remaining four age groups were below 1, indicating a negligible risk within these populations. However, the assessment of lifetime non-carcinogenic risk indicated that after 30 years of continuous exposure, there was a significant increase in the risk of non-carcinogenic effects. Previous studies have indicated that infants, toddlers, and children are more sensitive to POPs compared with adolescents and adults [10,85,86]. Consequently, they are more susceptible to the threats of PCB pollutants and need to receive more attention.

## 5. Conclusions

A comprehensive investigation of PCB pollution in surface waters was conducted for the first time in the southeastern TP. This study found that the composition of PCBs was predominated by PCB52 and PCB81, which exhibited widespread distribution. The ∑_3_PCB concentrations ranged from 738 ng/L to 1914 ng/L, with a mean value of 1058 ng/L, which is relatively higher than the values reported in other regions around the TP. Anthropogenic activities, LRAT, and glacial melting were the primary driving factors affecting PCB distribution. The riverine locations in proximity to the village and town exhibited elevated levels of pollution. In the Meili Snow Mountains area, PCBs originated from combustion, reductive dechlorination processes, commercial PCB mixtures, and electronic device disposal. An ecological risk assessment indicated severe pollution of PCBs, with predicted adverse ecotoxicological effects. Notably, PCBs posed non-carcinogenic risks to adults, and no cancer risk to five age groups. This study represents a preliminary investigation into the occurrence and composition of PCBs in the aquatic environment of the TP, and highlights the importance of gaining a deeper understanding of the health and ecological risks of PCBs in the temperate glacial watershed. However, there is a lack of data on PCB levels in the atmosphere and river sediments, which can provide a more comprehensive understanding of the sources of PCB pollution. Furthermore, PCBs’ ubiquitous occurrence and elevated levels, as well as their potential health risks to residents, require further investigation over a long period in the future to achieve a more comprehensive and objective understanding of the ecological and health risks in the region, which is crucial for the development of effective protective measures.

## Figures and Tables

**Table 1:** Exposure evaluation parameter values.

Parameter	Meaning	Unit	Selected Values				
			Infants	Toddlers	Children	Adolescents	Adults
IR	Ingestion rate of water	L/d	0.911	0.861	1.28	1.414	1.85
EF	Exposure frequency	Ds/yr	365				
ED	Exposure duration	Yrs	1	2	8	16	40
BW	Body weight	kg	6.8	12.6	24.1	51.1	63.1
AT	Average time for carcinogenic/non-carcinogenic effect	Ds	25,550/10,950				

## Data Availability

The original contributions presented in this study are included in the article/Supplementary Materials. Further inquiries can be directed towards the corresponding author(s).

## Supplementary Materials

The following supporting information can be downloaded at: https://www.mdpi.com/article/10.3390/toxics13050391/s1, Table S1: Detailed information on the water samples collected from the Meili Snow Mountains in the southeastern Tibetan Plateau; Table S2: The details of the recovery and precision of PCBs; Table S3: The concentrations of individual PCBs and ∑_3_PCBs (ng/L) for every sampling point in the Meili Snow Mountains, southeastern Tibetan Plateau; Table S4: The mean ∑PCBs (pg/m^3^) in air samples from different regions of the Tibetan Plateau; Table S5: The mean ∑PCBs (pg/g) in soil samples from different regions of the Tibetan Plateau; Table S6: The mean ∑PCBs (ng/L) in water samples from different regions around the Tibetan Plateau.

## Abbreviations

PCBs	Polychlorinated biphenyls
POPs	Persistent organic pollutants
TP	Tibetan Plateau
US EPA	United States Environmental Protection Agency
LRAT	Long-range atmospheric transport
TEQs	Toxic equivalency concentrations
DL PCBs	Dioxin-like PCBs
non-DL PCBs	Non-dioxin-like PCBs
LoD	Limit of detection
HQ	Hazard quotient
ILCR	Incremental lifetime cancer risk
TEF	Toxic equivalency factor
CSF	Carcinogenic slope factor
NOAA	National Oceanic and Atmospheric Administration
HYSPLIT	Hybrid Single-Particle Lagrangian Integrated Trajectory
CMC	Criterion maximum concentration
CCC	Criterion continuous concentration
AhR	Aryl hydrocarbon receptor
