# Supplementary material for: Glacial Waters Under Threat: Risk Assessment and Source Identification of Polychlorinated Biphenyls in Meili Snow Mountains, Southeastern Tibetan Plateau
Source: Toxics. 2025 May 13;13(5):391. doi: 10.3390/toxics13050391 (PMC12115771; doi:10.3390/toxics13050391)
Supplement: Supplementary file 1 [file toxics-13-00391-s001.zip › toxics-3588746-supplementary.pdf]

Glacial Waters Under Threat: Risk Assessment and Source Identification of Polychlorinated Biphenyls in Meli Snow Mountains, Southeastern Tibetan Plateau

Huawei Zhang[a*], Yan Yao[b], Xinyu Wen[b*], Rui Zhang[a], Rui Liu[b]

[a] Faculty of Geography, Yunnan Normal University, Kunming 650500, China; gtjdxl@outlook.com(R.Z.)

[b] College of Geography and Land Engineering, Yuxi Normal University, Yuxi 653100, China; yaoyan@yxnu.edu.cn (Y.Y.), lr870410@yxnu.edu.cn (R.L.)

*Corresponding author: hwzhang@ynnu.edu.cn, Tel.: +86-18388153750; wenxinyu@yxnu.edu.cn

Table S1. Detailed information of water samples collected from Meili Snow Mountains in the southeastern of Tibetan Plateau

Watershed	No.	Latitude(N)	Longitude(E)	Altitude(m)	Type
Qunatong River	gs-1	28.46066	98.59577	3829	Glacier meltwater
	gs-2	28.46090	98.59502	3847.2	Glacier meltwater
	gs-3	28.46613	98.61479	3936.1	Glacier meltwater
	gs-4	28.46135	98.60872	3898.7	Glacier meltwater
	gs-5	28.48100	98.57800	3763.3	Glacier meltwater
	gs-6	28.48593	98.56980	3749.9	River water
Pojun River	pj-1	28.51964	98.65910	3985.6	Glacier meltwater
	pj-2	28.51971	98.65840	3992.7	Glacier meltwater
	pj-3	28.52813	98.64638	4541.5	Glacier meltwater
	pj-4	28.59302	98.70870	2919.9	River water
Mingyong River	my-1	28.45306	98.75836	2818.2	Glacier meltwater
	my-2	28.46013	98.77366	2549	River water
Sinong River	sn-1	28.47866	98.73947	3431.9	Glacier meltwater
	sn-2	28.47922	98.73892	3443.8	Glacier meltwater
	sn-3	28.48909	98.7709	2678.5	River water
Yubeng River	yb-1	28.40846	98.74364	3882.8	Glacier meltwater
	yb-2	28.40903	98.74351	3860.7	Glacier meltwater
	yb-3	28.39987	98.77483	3242.9	River water

Table S2. The detail of the recovery and precision of PCBs

Category	Detection Times	Recovery (%)	Standard Deviation (%)
PCB28	6	79.3	4.19
PCB52	6	85.7	1.24
PCB101	6	79.4	3.36
PCB81	6	85.3	3.73
PCB77	6	100.1	1.77
PCB123	6	92.3	3.77
PCB118	6	94	2.80
PCB114	6	88.7	4.48
PCB138	6	82.1	1.29
PCB105	6	89.1	4.97
PCB153	6	87.9	2.25
PCB126	6	86.5	2.16
PCB167	6	91.2	2.15
PCB156	6	100.5	4.74
PCB157	6	84.9	2.40
PCB180	6	98.1	1.32
PCB169	6	90.7	3.50
PCB189	6	93.0	2.35

Table S3. Concentration of individual PCBs and ∑PCBs (ng/L) for every sampling point in Meili Snow Mountains, southeastern Tibetan Plateau

No.	PCB 28	PCB 52	PCB 77	PCB 81	PCB 101	PCB 105	PCB 114	PCB 118	PCB 123	PCB 126	PCB 138	PCB 153	PCB 156	PCB 157	PCB 167	PCB 169	PCB 180	PCB 189	∑PCBs
gs-1	<2.0	374	<2.0	568	<2.0	<2.0	<2.0	<2.0	<2.0	<2.0	<2.0	<2.0	<2.0	<2.0	<2.0	<2.0	<2.0	<2.0	942
gs-2	<2.0	410	<2.0	762	<2.0	<2.0	<2.0	<2.0	<2.0	<2.0	<2.0	<2.0	<2.0	<2.0	<2.0	<2.0	<2.0	<2.0	1172
gs-3	<2.0	383	<2.0	524	<2.0	<2.0	<2.0	<2.0	<2.0	<2.0	<2.0	<2.0	<2.0	<2.0	<2.0	<2.0	<2.0	<2.0	907
gs-4	<2.0	444	<2.0	384	<2.0	<2.0	<2.0	<2.0	<2.0	<2.0	<2.0	<2.0	<2.0	<2.0	<2.0	<2.0	<2.0	<2.0	828
gs-5	<2.0	652	<2.0	276	<2.0	<2.0	<2.0	<2.0	<2.0	<2.0	<2.0	<2.0	<2.0	<2.0	<2.0	<2.0	<2.0	<2.0	928
gs-6	<2.0	483	<2.0	475	<2.0	<2.0	<2.0	<2.0	<2.0	<2.0	<2.0	<2.0	<2.0	<2.0	<2.0	<2.0	<2.0	<2.0	958
pj-1	<2.0	475	<2.0	455	<2.0	<2.0	<2.0	<2.0	<2.0	<2.0	<2.0	<2.0	<2.0	<2.0	<2.0	<2.0	<2.0	<2.0	930
pj-2	<2.0	632	<2.0	295	<2.0	<2.0	<2.0	<2.0	<2.0	<2.0	<2.0	<2.0	<2.0	<2.0	<2.0	<2.0	<2.0	<2.0	927
pj-3	<2.0	544	<2.0	383	<2.0	<2.0	<2.0	<2.0	<2.0	<2.0	<2.0	<2.0	<2.0	<2.0	<2.0	<2.0	<2.0	<2.0	927
pj-4	<2.0	479	<2.0	521	<2.0	688	<2.0	<2.0	<2.0	<2.0	<2.0	<2.0	<2.0	<2.0	<2.0	<2.0	<2.0	<2.0	1688
my-1	<2.0	583	<2.0	402	<2.0	<2.0	<2.0	<2.0	<2.0	<2.0	<2.0	<2.0	<2.0	<2.0	<2.0	<2.0	<2.0	<2.0	985
my-2	<2.0	611	<2.0	578	<2.0	725	<2.0	<2.0	<2.0	<2.0	<2.0	<2.0	<2.0	<2.0	<2.0	<2.0	<2.0	<2.0	1914
sn-1	<2.0	343	<2.0	552	<2.0	<2.0	<2.0	<2.0	<2.0	<2.0	<2.0	<2.0	<2.0	<2.0	<2.0	<2.0	<2.0	<2.0	895
sn-2	<2.0	397	<2.0	341	<2.0	<2.0	<2.0	<2.0	<2.0	<2.0	<2.0	<2.0	<2.0	<2.0	<2.0	<2.0	<2.0	<2.0	738
sn-3	<2.0	394	<2.0	482	<2.0	<2.0	<2.0	<2.0	<2.0	<2.0	<2.0	<2.0	<2.0	<2.0	<2.0	<2.0	<2.0	<2.0	876
yb-1	<2.0	588	<2.0	482	<2.0	<2.0	<2.0	<2.0	<2.0	<2.0	<2.0	<2.0	<2.0	<2.0	<2.0	<2.0	<2.0	<2.0	1070
yb-2	<2.0	512	<2.0	513	<2.0	<2.0	<2.0	<2.0	<2.0	<2.0	<2.0	<2.0	<2.0	<2.0	<2.0	<2.0	<2.0	<2.0	1025
yb-3	<2.0	332	<2.0	341	<2.0	662	<2.0	<2.0	<2.0	<2.0	<2.0	<2.0	<2.0	<2.0	<2.0	<2.0	<2.0	<2.0	1335

Table S4. Mean ∑PCBs (pg/m^3) in air samples from different regions of the Tibetan Plateau

Longitude	latitude	Region	Location or description	Congeners	Mean	Sampling time	Reference
94.91	36.39	Qinghai	Golmud	15 PCBs	4.2	2007-2008	[37]
97.03	33.02	Qinghai	Yushu	15 PCBs	5.0	2007-2008	[37]
80.09	32.50	Tibet	Gar	15 PCBs	5.0	2007-2008	[37]
84.06	32.31	Tibet	Gaerze	15 PCBs	3.9	2007-2008	[37]
85.23	29.33	Tibet	Saga	15 PCBs	3.2	2007-2008	[37]
86.95	28.36	Tibet	Everest	15 PCBs	4.2	2007-2008	[37]
88.89	29.25	Tibet	Xikaze	15 PCBs	7.1	2007-2008	[37]
91.03	29.65	Tibet	Lhasa	15 PCBs	4.4	2007-2008	[37]
91.98	31.42	Tibet	Naqu	15 PCBs	3.0	2007-2008	[37]
93.24	29.88	Tibet	GBJD	15 PCBs	4.9	2007-2008	[37]
95.77	29.86	Tibet	Bomi	15 PCBs	8.2	2007-2008	[37]
96.91	29.37	Tibet	Rawu	15 PCBs	6.0	2007-2008	[37]
97.14	31.15	Tibet	Qamdo	15 PCBs	7.3	2007-2008	[37]
99.05	37.47	Qinghai	Tianjun county, summer	12 PCBs	0.58	2010	[38]
101.79	36.60	Qinghai	Xining City, summer	12 PCBs	0.52	2010	[38]

79.70	33.39	Tibet	Ngari	6 PCBs	2.55	—	[39]
75.04	38.41	Xinjiang	Muztagh Ata	6 PCBs	9.7	—	[39]
90.99	30.77	Tibet	Nam Co lake	6 PCBs	2.5	2012-2014	[26]
94.65	29.62	Tibet	Western slope in the Shergyla Mountain	25 PCBs	0.86	2010-2011	[40]
102	29.58	Tibet	Mt. Gongga	—	47	2012	[41]
98.48	24.95	Yunnan	Tengchong Mountain	11 PCBs	53.6	2005-2006	[42
100.21	26.90	Yunnan	Lijiang's atmosphere	6 PCBs	1.6	2009-2013	[43]

Table S5. Mean ∑PCBs (pg/g) in soil samples from different regions of the Tibetan Plateau

Longitude	latitude	Region	Location or description	Congeners	Mean	Sampling time	Reference
102.39	27.58	Sichuan	Remote sites	16 PCBs	0.52	2012-2013	[44]
102	29.57	Sichuan	Remote sites	16 PCBs	0.668	2012-2013	[44]
103.18	33.44	Sichuan	Ruoergai, grassland and wetland	6 PCBs	0.94	2011	[45]
91.09	29.65	Tibet	—	18 PCBs	0.015	2012	[46]
90.59	30.57	Tibet	—	18 PCBs	0.019	2012	[46]
89.64	29.35	Tibet	—	18 PCBs	0.039	2012	[46]
80.09	32.50	Tibet	Gar: Sub-alpine desert soils	8 PCBs	0.193	2007	[47]
91.90	32.37	Tibet	Naqu: Alpine meadow soils	8 PCBs	0.198	2007	[47]
81.60	34.60	Tibet	Hoh Xil: Alpine desert soils	8 PCBs	0.121	2007	[47]
88.63	33.82	Tibet	Linge co: Alpine steppe soils	8 PCBs	0.257	2007	[47]
85.23	29.33	Tibet	Zhongba: mountain shrubby steppe soils	8 PCBs	0.302	2007	[47]
95.77	29.86	Tibet	Bomi: Alpine-burozems	8 PCBs	0.292	2007	[47]
96.48	32.51	Tibet	Nangqen: Sub-alpine scrub-meadow soils	8 PCBs	0.596	2007	[47]
94.33	29.33	Tibet	Yarlung Tsangpo Valley	18 PCBs	0.057	2010-2011	[48]

Table S6. Mean ∑PCBs (ng/L) in water samples from different regions around the Tibetan Plateau

Sample Region	Abbr. Sample Region	Congeners	Mean	Range	Sample types	Sampling time	Reference
Rongbuk Glacier	RG	7 PCBs	0.016	0.003-0.048	snowpack	2004-2005	[49]
Dasuopu Glacier	DG	7 PCBs	0.2	0.09-0.34	firn core	2005	[50]
Chenab River	CR	32 PCBs	5.72	0.2-27.5	surface water	2013	[51]
Ganges River	GR	6 PCBs	0.04	0.017-0.064	surface water	2013	[52]
Hengduan Mountains	HM	18 PCBs	670	ND-1421	surface water	2022	[10]
Qilian Mountains	QM	18 PCBs	138	ND-211	surface water	2022	[10]
Meli Snow Mountains	MLSM	18 PCBs	1058	738-1914	surface water	2023	This study
